# Impact of Aggregate Grain Size on ASR-Induced Expansion

**DOI:** 10.3390/ma16247506

**Published:** 2023-12-05

**Authors:** Justyna Zapała-Sławeta

**Affiliations:** Faculty of Civil Engineering and Architecture, Kielce University of Technology, Al. Tysiąclecia Państwa Polskiego 7, 25-314 Kielce, Poland; jzapala@tu.kielce.pl

**Keywords:** alkali–silica reaction, reactive aggregate, grain size, corrosion, ASR gel, microstructure degradation, computed tomography, SEM-EDS

## Abstract

Alkali–silica reaction (ASR) is a sequence of complex chemical processes, resulting in the formation of alkali silica gels with high swelling ability. ASR leads to the expansion of concrete and the degradation of its microstructure. The susceptibility of aggregates to alkali reaction depends, among other factors, on the type and origin of the aggregate, the presence of reactive forms of silica, the mineral composition, and the geometric properties of the aggregate, such as shape and grain size. This study aimed to investigate the impact of the grain size of polymineral post-glacial gravel aggregate, originating from the northern regions of Poland, on its susceptibility to ASR. The expansion of mortars made from polymineral aggregate and the cracking of grains and cement matrix due to the occurring reactions were analyzed. Based on the conducted research, it was observed that the expansion of mortars depends on the grain size of the aggregate. It was demonstrated that the fraction of reactive aggregate generating the most significant elongation of mortars is in the range of 1.0–2.0 mm. The reaction of silica with alkalis continued until the depletion of reactive components in the aggregate. The relationship between the progress of corrosive processes and the grain size of the aggregate was evident in the form of different linear elongation increments of mortars over time. The expansion of mortars was caused by the swelling ASR gel, inducing stress in the grain and the surrounding cementitious paste.

## 1. Introduction

Aggregates constitute approximately 70–75% of the volume of concrete. The quality of the used aggregate is determined by normative requirements concerning geometric and physical properties, including durability [1]. Aggregates containing certain forms of amorphous, low-crystalline, or microcrystalline silica that exhibit potential reactivity with alkalis, and the products of these reactions, are alkali silica gels with swelling properties [2]. The use of potentially reactive aggregates in concrete is limited and requires preventive measures such as mineral additives for concrete or cement and chemical admixtures such as lithium compounds [3,4,5,6,7]. The macroscopic effect of these reactions, reflecting changes in the microstructure, is the expansion of mortars and concrete made with reactive aggregates.

The reactivity of aggregates mainly depends on the petrographic composition. Reactive minerals are classified into fast to normal reactive (5–20 years), slow/late reactive (above 15 to 20 years), and non-reactive aggregates [8]. The thermodynamic stability of various forms of silica decreases as the degree of microstructural disturbance increases. Fast-reacting minerals include opal, tridymite, cristobalite, and chalcedony, which are metastable forms of silica, while aggregates containing strained quartz react slowly [9,10]. However, the relationship between the content of the reactive constituent in the aggregate and the expansion of the concrete made with the aggregate is not always direct. Numerous studies indicate that expansion increases with the content of the reactive constituent only up to a certain level (pessimum), beyond which it decreases [2,11,12,13]. For slow-reacting aggregates, the pessimum effect may not occur, and the highest expansion occurs in the case of high-aggregate contents, unlike fast-reacting aggregates, where the pessimum effect occurs for an aggregate content below 10% [14].

The grain size of the aggregate also affects its ability to induce a harmful reaction [15]. Both fine and coarse aggregates can induce destructive ASR, but the aggregate size affects the magnitude of expansion [16,17]. In many cases, finer aggregate grains reacted faster than coarser ones, affecting the degree of expansion [15,18]. Vivian noted maximum expansion values when using opal aggregate with grain sizes of 0.07–0.85 mm, indicating that smaller and larger grains lead to reduced expansion [19]. Other researchers confirmed the increase in expansion with a decrease in grain size, but only up to 0.02 mm [15]. Poyet [20], studying highly reactive silica, found that very fine fractions did not cause expansion, which was also confirmed by Cyr et al. when studying fine aggregates of various types [21]. The behavior of the aggregate was linked to the pozzolanic reaction of fine reactive particles. Multon et al. investigated siliceous limestone aggregate and demonstrated that a particle size fraction of 1.18–2.36 mm showed the largest expansion [22]. Similarly, Dunant and Scrivener found an Alpine chlorite schist fraction of 2–4 mm to be the most severe threat to concrete [17]. Thus, it should be emphasized that aggregate reactivity is particle size dependent, not necessarily in a predictable way, and the reasons for this phenomenon vary. As the literature indicates, different levels of reactivity of aggregate particles with alkalis may be due to the time required to diffuse OH^−^ ions to reactive minerals that may be located in non-reactive matrices with different structures. Also, the magnitude of stresses that cause reactive aggregate fracture depends on the aggregate size [17,22].

Concrete with larger aggregate grain sizes often exhibited delayed expansion. Nishibayashi and Yamura demonstrated that the expansion of concrete containing only coarse aggregate increased slowly but with aging. Using only fine reactive aggregate resulted in higher expansion in the initial period, followed by stabilization [23]. Interestingly, the authors observed reduced expansion in concrete when using both fine and coarse reactive aggregate. Wigum and Lindgard [24] also indicated that slowly reacting coarse aggregate from Norway is more harmful than fine aggregate, so Norwegian recommendations consider the possibility of increased expansion over time, multiplying the values of linear expansion obtained in accelerated tests by a factor greater than one [25,26,27].

Analyzing the influence of the aggregate grain size on the expansion generated by mortars and concrete affected by ASR, it is essential to consider the reaction rate of silica with alkalis, which is higher for smaller aggregate particles, as well as the overall long-term expansion, which may be greater for aggregates with larger grain sizes. The situation may be more complex for polymineral aggregates containing silica with different reaction kinetics with hydroxide ions and due to the distribution of reactive minerals in the non-reactive matrix of the aggregate, thereby varying the availability of aggressive ions to silica. 

Polymineral gravels are widely used for concrete, including buildings and engineering structures in various classes of concrete structures exposed to variable environmental conditions [28,29]. As Brandt points out, in countries with no volcanic activity, such as Poland, certain postglacial aggregates can have harmful ASR effects on concrete structures, becoming apparent from a few months to years after construction [30]. The properties of aggregates obtained from crushing fully rounded postglacial aggregates (pebbles) often match those of crushed aggregates from solid magmatic rocks [31]. Therefore, the need arises to expand the study of polymineral aggregates to cover the factors contributing to their reactivity and failure mechanism, such as mineral composition, gradation, and the distribution of reactive constituents in the rock. 

This study presents the influence of various fractions of polymineral gravel aggregate on ASR-induced expansion. The alkali reactivity of individual fractions of mechanically crushed aggregate was analyzed using the ASTM C1260 method. The expansion of mortars made entirely with reactive aggregate was compared with the linear changes in mortars induced by the reaction of individual fractions of polymineral aggregate with alkalis. The obtained results were related to the observation of the microstructure using computed tomography and scanning electron microscopy with the determination of compositions of reaction product. Based on the obtained research results, a likely course of corrosive processes due to the reaction of natural post-glacial aggregate with alkalis was proposed. 

## 2. Materials and Methods

The alkaline reactivity analysis was conducted on post-glacial polymineral gravel aggregate from northern regions of Poland. Previous studies have categorized this aggregate as reactive [32]. It was shown that the expansion of mortars and concretes made with this aggregate exceeds the aggregate reactivity thresholds, i.e., the expansion limit of 0.20% after 14 days (ASTM C1260) and 0.04% after 365 days (according to ASTM 1293) [33]. The petrographic composition of the aggregate, indicating the percentage of reactive rocks, is presented in Table 1. Petrographic analysis of the studied aggregate indicated that three minerals are mainly responsible for its reactivity: chalcedony, opal and strained quartz. These reactive constituents were identified in metamorphic quartz pyroxene shale containing opal binder, quartz–glauconite sandstone with claycarbonate binder including some chalcedony, and quartz grains in feldspar–biotite granite.

Grains with sizes of 8–16 mm were mechanically crushed, resulting in aggregate with a grain size of 0.125–4.00 mm. The aggregate was then passed through a set of sieves, obtaining five fractions: 0.125–0.25 mm, 0.25–0.5 mm, 0.5–1 mm, 1–2 mm, and 2.0–4.0 mm. Non-reactive limestone aggregate was used, which was crushed to the same dimensions as the polymineral aggregate to replace individual fractions of it and assess their reactivity. Portland cement CEM I 42.5 R was used as the binder, with its composition presented in Table 2 and an alkali content of 0.85% Na_2_O_e_.

The accelerated method for assessing the potential reactivity of the aggregate was used, following the ASTM C1260 standard [35]. The ratio of aggregate to cement was constant at 2.25, with a *w*/*c* ratio of 0.47. The proportions of aggregates in the mortars are presented in Table 3. Three 25 × 25 × 250 mm bars were made for each of the mortar series, which then matured for 24 h under laboratory conditions. Subsequently, the samples were stored for 24 h in water at a temperature of 80 °C, after which the so-called initial measurements of the samples were taken using the Graff Kaufman apparatus with an accuracy of 0.005 mm. The mortar bars were then transferred to a 1 M NaOH solution and stored for 14 days, with their lengths were measured daily. The expansion value of the mortars in a given series was determined as the average of measurements from the 3 bars.

The degree of mortar damage was assessed visually and using computed tomography and scanning electron microscopy. These examinations were conducted after the completion of the test, i.e., after 14 days of storing the mortars under corrosive conditions. A Nikon XT H 225 ST CT tomography scanner (Nikon Metrology, Surbiton, Surrey, UK) was used in the study, equipped with a tube that generates a beam of radiation at a max. voltage of 225 kV and a power of 450 W. Scans were performed at a voltage of 220 kV and a current intensity of 77 µA. These values were selected experimentally by scanning the sample several times to obtain the best possible parameters for cement composite material. For each sample, 4500 2D images were acquired, with an exposure time of 250 µs for each image. After compiling the two-dimensional images, a three-dimensional model was created using the CT Pro 3D software (version XT 5.4). Subsequently, the 3D model was analyzed using VG Studio Max 3.4 software.

Computed tomography, as a non-destructive technique, allows for the observation of in situ reactions. Observations of the microstructure under a scanning electron microscope required proper preparation of the samples. For this purpose, after 14 days of testing, samples measuring 25 × 25 × 25 mm were taken from the middle parts of the mortar beams, cut to the size of 25 × 25 × 10 mm and subjected to grinding and polishing procedures. For the observation of the microstructure and chemical composition analysis of the products, SEM-EDS was utilized. This was performed using a scanning electron microscope equipped with an X-ray analyzer (SEM; FEI Company, Hillsboro, OR, USA). Observations were conducted in Backscattered Electron (BSE) mode at a voltage of 15 keV. The composition of the reaction products is presented as the average of 3 measurements at points close to each other. 

## 3. Results and Discussion

### 3.1. ASR Expansion

**Figure 1:**
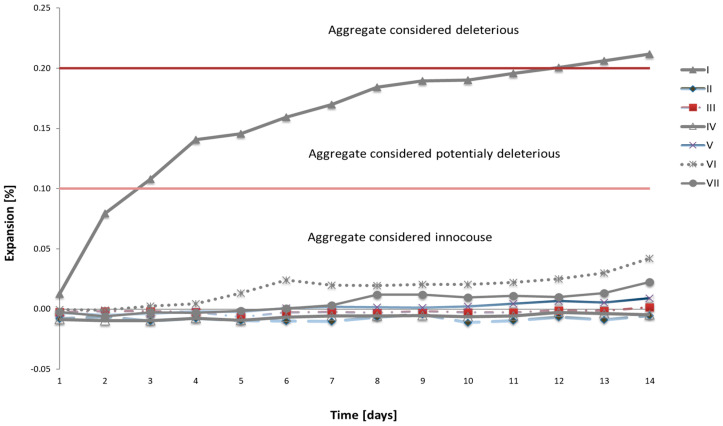
The expansion of mortars with various fractions and percentages of polymineral aggregate. The red lines represent the boundaries of potential reactivity and reactivity of the aggregate, following ASTM C1260.

The results of the expansion tests on mortars made with reactive post-glacial aggregate and mortars made with limestone aggregate, according to the formulations presented in Table 2, are shown in Figure 1. After 14 days of testing, the expansion of mortars with polymineral aggregate is 0.21%, confirming its moderate reactivity. Limestone aggregate is alkali-neutral, as evidenced by the absence of linear changes in the mortars during the tested period. Mortar series III–VII, containing only one of the fractions of reactive aggregate in the mix, exhibit varying expansion values. The results indicate that mortars labeled as “III” and “IV”, containing 15% of the 0.125–0.25 mm fraction and 25% of the 0.2–0.5 mm fraction, respectively, show no volume changes. The expansion of mortars increases with the size of the aggregate, but the values remain small. Mortars with 25% of the 0.5–1.0 mm fraction and 10% of the 2.0–4.0 mm fraction show small and comparable linear changes. The highest linear elongation values were recorded in mortars with 25% of the 1.0–2.0 mm fraction (series VII). None of the individual fractions of reactive aggregate generate expansion exceeding the threshold of potential reactivity, which is 0.1%, after 14 days of testing. It was also observed that the expansion of mortars with single fractions of polymineral aggregate with grain sizes of 1–2 mm and 2–4 mm is delayed compared to mortars made entirely of this aggregate. The obtained results confirm literature reports indicating the possibility of delaying harmful alkali reactions with larger aggregate grains [15,23].

**Figure 2:**
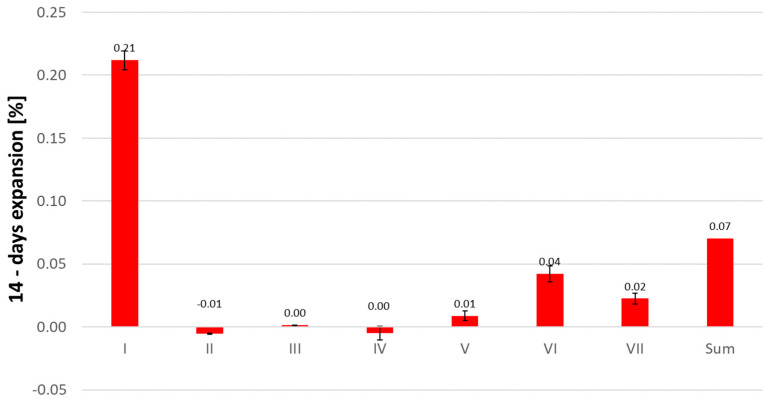
Comparison of the ASR-expansion of mortars containing individual size fraction of reactive aggregate and non-reactive limestone.

In Figure 2, the expansion of mortars containing individual fractions of reactive aggregate is presented, and their sum is compared to the expansion values of mortars containing 100% tested aggregate. The total expansion of mortars containing individual fractions of reactive aggregate and non-reactive limestone is three times smaller than the expansion of mortars containing only polymineral post-glacial aggregate. Ramyar and others, studying similar reactive systems using individual fractions of reactive natural river aggregate and non-reactive limestone, found that the total expansion of mortars containing individual fractions of aggregate significantly exceeded the expansion of mortars made entirely from reactive aggregate, especially for mechanically crushed aggregates [36]. This phenomenon can be explained by the barrier effect resulting from the creation of numerous corrosion centers in mortars made entirely from reactive aggregate [14]. However, in the systems under consideration, the mechanism appears to be different. Dunant and Scrivener, citing Reinhardt and Mielich, emphasized the significance of cracking mechanics influenced by the forces exerted by aggregates on the surrounding environment and the varying lengths of propagating cracks within the aggregate [17,37]. They demonstrated that there is a critical crack length in grains, dependent on their dimensions, leading to aggregate damage. When cracking reaches the cement paste, further propagation depends on the properties of the paste, meaning that the expansion rate in later stages is not influenced by aggregate size. The expansion increases due to the cracking of the cement matrix, and the reactive components become depleted. Additionally, as indicated by Dunant and Scrivener, the expansion of mortars with individual fractions of aggregate is smaller because the microstructure of these mortars is different [17]. In mortars with 100% reactive aggregate, the cement paste is under stress generated by the swelling grains. The expansion of mortars with 1–2 mm fraction aggregate is greater than that of mortars with 2–4 mm fraction aggregate, which can also be attributed to the higher solubility of silica in the 1–2 mm fraction compared to the 2–4 mm fraction, as demonstrated in previous research [38]. As the aggregate grain size decreases, its reactivity may increase due to the larger surface area of fine aggregate, resulting in faster reactions involving reactive silica dissolution. It is possible that highly fragmented silica behaves like a pozzolanic material, which is a favorable phenomenon that reduces the risk of harmful reactions [16,39].

### 3.2. Macroscopic and Microscopic Examination

**Figure 3:**
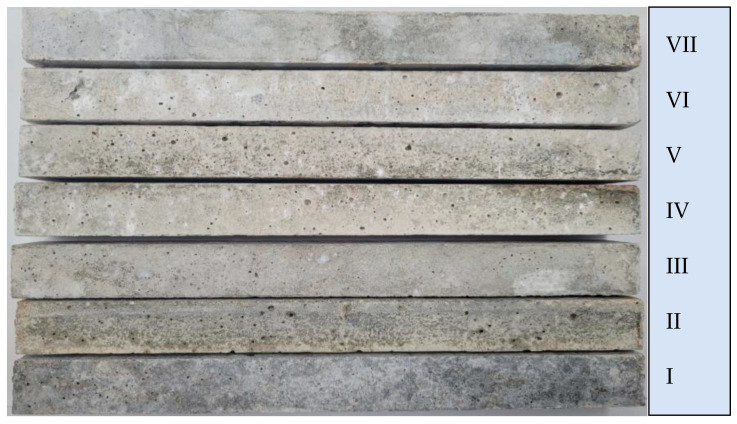
Visual assessment of mortar bars containing individual size fractions of reactive aggregate and non-reactive limestone.

The macroscopic assessment of mortars containing various fractions of reactive aggregate indicates the formation of pop outs, leakage of alkali gels, and fine cracks, especially in mortars V (fraction 0.5–1 mm), VI (fraction 1–2 mm), and VII (fraction 2–4 mm). In the bars from mortars containing finer fractions of aggregate, leakage of ASR gels was observed, while no other macroscopic signs of reaction were revealed (Figure 3).

**Figure 4:**
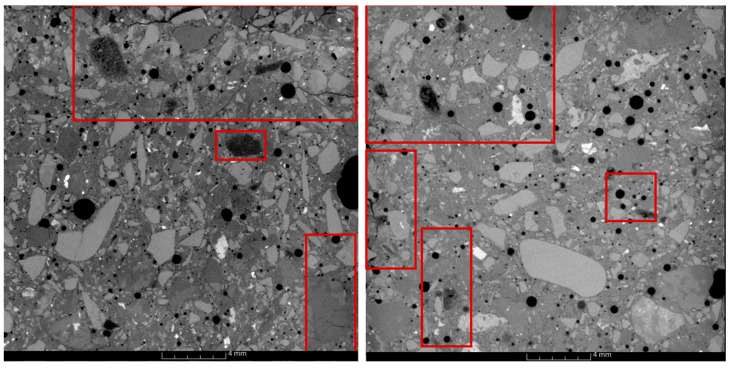
Example cross-sections of mortars with reactive aggregate obtained by computed tomography with red-framed cracks and corrosion centers.

The microstructure analysis of mortars was performed based on images obtained using computed tomography. Samples of mortars containing 100% reactive aggregate were evaluated. Selected mortar images are presented in Figure 4. A tomographic image is created by assigning a grayscale value to each voxel. The grayscale value is associated with the attenuation of X-ray penetration through the examined object [40]. Objects with lower density, including air pores, are visible in the images as darker areas, while objects with higher density appear as brighter areas. 

The images of mortar structures demonstrate that both fine and larger grains undergo corrosive processes. Corroded grains are visible as darker areas, and the degree of their overreaction is significant. Cracks are visible within the volume of fine and coarse reactive grains, empty spaces within the volume of larger grains, cracks in the cement paste, and accumulation of ASR products in some air voids. The tomographic images show that gels in the air voids induce stress in the cement paste, leading to its cracking. The obtained results align with macroscopic observations, where alkali gel leaks were detected in all mortar series, indicating ongoing corrosive processes.

**Figure 5:**
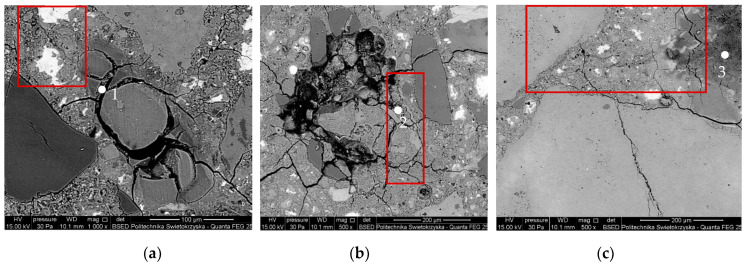

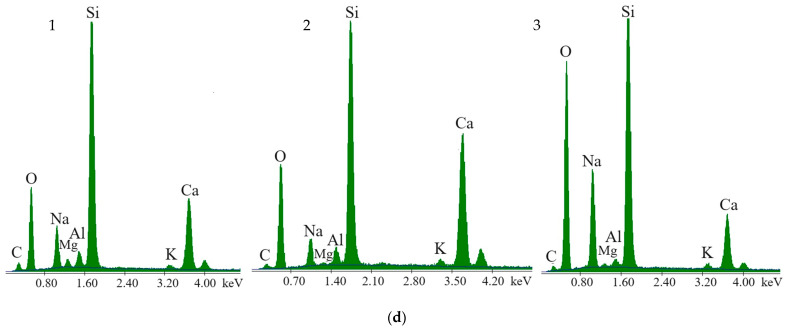
Microstructure of mortars with reactive gravel aggregate: (**a**–**c**) cracked grains, (**d**) X-ray microanalysis at points 1–3. Red frames mark ASR gels in the cement paste and the ITZ (interfacial transition zone).

Microstructural observations of mortars with reactive aggregate, conducted under a scanning electron microscope, revealed damage to the grains due to reactions with sodium and potassium hydroxides. This is confirmed by chemical composition analyses in microareas, indicating the formation of sodium–potassium–calcium silica gel. It should be noted that post-glacial gravel aggregate grains react differently, either in some areas of their volume or throughout their volume (Figure 5a–c), generating various stresses, as evidenced by different degrees of grain degradation. Gels were observed moving into the cement paste, filling the cracks (Figure 5a,c), and generating stresses within the reactive grain volume (Figure 5a,b). The presented microstructural images indicate a high level of advancement in the corrosive processes in the polymineral aggregate, regardless of grain size.

The mechanism for initiating expansion due to ASR, based on current knowledge, relies on the reactive envelope model proposed by Ichikawa and the random gel package model proposed by Dunant [17,41,42]. Both models assume that the expansion pressure occurs at the reaction site within a limited area, which is the grain itself. This means that the expansion pressure increases within the aggregate, leading to cracks in the grain and their propagation into the cement paste. 

Gels formed in reactive grains show different chemical compositions and can generate different swelling pressures [43]. ASR gel may not generate expansion by filling the cracks in the cement paste but solidifies due to the exchange of Na^+^ and K^+^ ions for Ca^2+^ ions, acting as an obstacle preventing further gel accumulation in the cracks. This results in increased pressure and leads to the further propagation or widening of cracks. Therefore, macroscopic expansion growth depends on changes in the amount of gel produced over time, inducing pressure. In the case of gravel aggregate, whose reactivity was examined using the accelerated method, the ASR rims were not observed around the grains, but the grain reacted in specific areas of its volume or throughout its volume, depending on the distribution and content of reactive silica, until it was depleted. This suggests that under the given conditions, the mechanism of the corrosive processes is similar to that proposed by Dunant and Scrivener [17,42]. 

Studying the course of corrosive processes in gravel aggregates is complex due to their polymineral nature. The distribution of cracks in the aggregate and cement paste has a significant impact on the macroscopic damage caused by ASR. Factors influencing the observed method and degree of microstructural damage to mortars include grain texture, distribution of reactive silica within the grain, defects, shape, aggregate fragmentation, and its surroundings. Therefore, cracks induced by ASR change under the influence of internal and external factors. The examined gravel contains several reactive rocks that react differently, with varied textures, in which reactive silica is present in the matrix of non-reactive rocks or the matrix itself is reactive. The fragmentation of the aggregate can increase the availability of aggressive ions to silica, accelerating its reaction, while larger grains probably react more slowly. As Czapik pointed out, in an analysis of the reaction of alkalis with polymineral aggregate, that the migration of alkalis in the hardened cement paste and aggregate matrix should be considered [34]. One should not overlook the changes in gel properties caused by the exchange of Na^+^ and K^+^ ions for Ca^2+^ ions, depending on the diffusion rate of these ions.

## 4. Conclusions

The presented research results investigated the influence of the natural post-glacial gravel aggregate’s particle size on the expansion caused by ASR. Extended studies, including macro and microscopic observations allowed for a more comprehensive analysis of the obtained results. The following conclusions were drawn:

1. Post-glacial gravel aggregate exhibits moderate reactivity with alkalis, and the reaction, under the conditions specified in the ASTM C1260 standard method, i.e., storage in 1 M NaOH at T = 80 °C, occurs quickly.
2. The particle size of the aggregate affects the expansion generated by it in mortars. Grains of all aggregate fractions containing reactive silica react with alkalis, resulting in the formation of ASR gel, visible on the surface of mortars as white efflorescence and identified in cracked grains, propagating into the cement paste.
3. The most harmful for mortar durability turned out to be reactive aggregate grains of the 1–2 mm fraction. Fine aggregate with a particle size of 0.125–0.5 mm, when reacting with alkalis, did not cause expansion in the mortars made from them. Mortars with an aggregate particle size of 2–4 mm, despite evident signs of reaction visible in the form of efflorescence and surface pop outs, also showed low expansion. The reaction of larger aggregate grains is likely slower, as evidenced by the delayed expansion. Surface pop outs are likely the result of greater availability of alkaline solution to the reactive grain.
4. The cumulative expansion of mortars made from individual fractions of the tested aggregate is approximately three times smaller than the expansion of mortars containing only gravel aggregate. It is presumed that the smaller cumulative expansion of mortars containing individual fractions of reactive aggregate results from the properties of the cement paste, as indicated by Durant and Scrivener, i.e., in mortars made with 100% gravel aggregate, all reactive grains induce stresses on the cement paste, leading to its cracking and, thus, larger expansions. In mortars with individual fractions of reactive aggregate, the microstructure of the cement paste is different, i.e., not weakened by many corrosion centers, and therefore exhibits different mechanical properties.
5. X-ray micro-computed tomography (µCT) indicates that both fine and coarse particles undergo a harmful reaction with alkalis, leading to various degrees of deterioration. Differences were noted in the degree of particle reaction and cracking due to the occurring corrosion processes, which was also confirmed by scanning electron microscopy. Significant cracking of the cement matrix was also observed.
6. The reaction products cover the reactive aggregate surfaces or fill the cracks within the aggregate particles. ASR gels generate various swelling pressures that cause varying degrees of particle deterioration. The gels also run through the cement paste, causing cracking and partially filling the cracks.
7. ASR gel, partially filling the air voids in mortars made from reactive aggregate, generates pressure in the cement paste, leading to its cracking. Therefore, the amount of gel produced in mortars affects the increase in expansion. The studied reactive system is complex and requires further research, including determining the influence of the cement-to-reactive aggregate ratio and analyzing the rheological properties of gels.
8. The indicated influence of the aggregate particle size on mortar damage caused by ASR is not synonymous with the necessity of analyzing individual aggregate fractions in assessing its susceptibility to ASR. It should be noted that in the case of mechanically crushed aggregates, the presence of cracks in the grains that facilitate the access of aggressive ions to reactive silica can be significant. The results obtained indicate that when considering the aggregate’s reactivity, 100% of its content should be taken into account.

## Figures and Tables

**Table 1:** Petrographic composition of aggregate (%) [34].

Rock	Quartz–Glauconite Sandstone	Organodetritic Sparitic–Micritic Limestone	Metamorphic Quartz–Pyroxene Shale	Feldspar–Biotite Granite	Non-Reactive Components
Content	13.8	28.0	3.9	10.5	43.8

**Table 2:** Chemical composition of cement CEM I 42.5 R (%).

Material	SiO_2_	Al_2_O_3_	Fe_2_O_3_	CaO	MgO	SO_3_	K_2_O	Na_2_O	Na_2_O_e_
Cement	19.60	4.60	3.15	62.35	2.19	3.11	1.02	0.18	0.85

**Table 3:** Aggregate proportions in cement mortars.

Fraction Size (mm)	Fraction Content (%)	Sample Marking						
		I	II	III	IV	V	VI	VII
		Pure		Substitution Mixtures				
0.125–0.25	10	R	L	R	L	L	L	L
0.25–0.5	25	R	L	L	R	L	L	L
0.5–1	25	R	L	L	L	R	L	L
1–2	25	R	L	L	L	L	R	L
2–4	15	R	L	L	L	L	L	R

Note: L—non-reactive aggregate (limestone), R—reactive aggregate (post-glacial gravel aggregate).

## Data Availability

Data are contained within the article.
